# Developing a Comprehensive Pesticide Health Effects Tracking System for an Urban Setting: New York City’s Approach

**DOI:** 10.1289/ehp.7149

**Published:** 2004-08-03

**Authors:** Daniel E. Kass, Audrey L. Thier, Jessica Leighton, James E. Cone, Nancy L. Jeffery

**Affiliations:** ^1^New York City Department of Health and Mental Hygiene, Bureau of Environmental Disease Prevention, New York, New York, USA; ^2^Consultant, Williamstown, Massachusetts, USA

**Keywords:** data linkage, epidemiology, New York City, pest control, pesticide use, poison control, surveillance

## Abstract

In recent years, there have been substantial investments and improvements in federal and state surveillance systems to track the health effects from pesticide exposure. These surveillance systems help to identify risk factors for occupational exposure to pesticides, patterns in poisonings, clusters of disease, and populations at risk of exposure from pesticide use. Data from pesticide use registries and recent epidemiologic evidence pointing to health risks from urban residential pesticide use make a strong case for understanding better the sale, application, and use of pesticides in cities. In this article, we describe plans for the development of a pesticide tracking system for New York City that will help to elucidate where and why pesticides are used, potential risks to varied populations, and the health consequences of their use. The results of an inventory of data sources are presented along with a description of their relevance to pesticide tracking. We also discuss practical, logistical, and methodologic difficulties of linking multiple secondary data sources with different levels of person, place, and time descriptors.

Pesticides are chemicals or other products used to kill, repel, or control pests and include insecticides, rodenticides, fungicides, and herbicides. Effectively assessing human health risks from pesticide use and exposure depends on the timely availability of data that describe how, where, when, by whom, why, what type, and in what quantities pesticides are used. A report by the Pew Environmental Health Commission (2000) has called for the development of state and local pilot environmental tracking systems to track, among other things, pesticide hazards and related health outcomes. This report helped to launch the national Environmental Public Health Tracking (EPHT) Program funded and organized by the U.S. Centers for Disease Control and Prevention (CDC). CDC defines environmental public health tracking as

the ongoing collection, integration, analysis, and interpretation of data about environmental hazards, exposure to environmental hazards, and human health effects potentially related to exposure to environmental hazards. (National Center for Environmental Health 2003)

Few examples exist where these three data domains—hazard, exposure, and health effects—are simultaneously tracked and linked, despite clear benefits of doing so (Council of State and Territorial Epidemiologists 1999).

In 2002 CDC awarded the New York City (NYC) Department of Health and Mental Hygiene (DOHMH) funding to develop its capacity to track environmental public health indicators. The following year, DOHMH was awarded an EPHT grant to develop a pilot pesticide tracking system for NYC. The goal of the EPHT program is to

demonstrate and evaluate methods for linking data from ongoing, existing health effects surveillance systems with data from existing surveillance/monitoring systems for human exposure and environmental hazards. (CDC 2003b)

Collectively, these programs are known at DOHMH as “Environmental Connections.” This article describes gaps in pesticide surveillance systems, a rationale for tracking pesticides in NYC, and NYC’s operational plan to create such a system.

## Existing Pesticide-Related Surveillance

EPHT defines hazard as a factor that may adversely affect health. Many sources of pesticide hazard data exist. For example, national databases exist that describe the names and classes of pesticides, their federal and state registration status, and their toxicologic properties, although there is no single database that consolidates information on both the acute and chronic health effects of pesticides. The U.S. Food and Drug Administration (FDA) samples domestic and foreign food products for pesticide residues and funds states for local food surveillance (FDA 2001). However, no national-scale surveillance system exists that makes data available on pesticide production, import/export, sale, application, or use (Donaldson et al. 2002). Absent such data, the U.S. Environmental Protection Agency (EPA), Department of Agriculture, and Geological Survey estimate annual pesticide use by linking manufacturer, industry, grower, and crop survey data. The industry and trade association production and sales data used for these estimates are available for purchase (Kegley et al., unpublished data). Several states estimate pesticide use through similar combination of sales, use, and crop surveys, but the utility of this approach is limited to characterizing agricultural use and may be incomplete and inadequate to characterize geographic areas smaller than states or even regions (Thier 1997).

Five states mandate some form of comprehensive pesticide use reporting (PUR) and sales. California’s regulations require that agricultural and commercial applicators and government institutions file pesticide use reports with the state. Agricultural reports must contain information on the identity, quantity, location, method, date, and other volume and acreage data of restricted-use applications. Reports for nonagricultural applications are less detailed because they are aggregated by month and county. In addition, all pesticide sales must be reported at the first point of sale (California Department of Pesticide Regulation 2000). California’s use and sales systems permit public access to line-item data. Massachusetts, Oregon, New Hampshire, and New York require PUR that includes agricultural, nonagricultural, building, and institutional applications, with varying degrees of experience and public access (Kegley et al., unpublished data).

Oregon is the only state that currently requires tracking of household pesticide use through point-of-sale reporting, although the state’s fiscal crisis has prevented Oregon from collecting use and sales reports (PURS-Oregon 2004). New York’s system is the best equipped among state PURs to characterize urban pesticide use because address, type, and quantity must be provided for all structural and rodent applications. However, the New York legislature imposed the most restrictive of the states’ public access requirements, permitting release of raw data only for human health research and only if approved by a stakeholder health science board (New York State Environmental Conservation Law 1997).

Data from many of these state PUR systems have been used to produce research papers, reports, and white papers explaining the purpose, distribution, and quantities of largely agricultural pesticides. Investigators have used California’s PUR data for ecologic studies examining Parkinson disease (Ritz and Yu 2000) and cancer incidence (Mills 1998). Less specific pesticide use data from state and federal agricultural agencies have also been used to identify a pattern of birth defects associated with certain pesticide use (Garry et al. 1996; Schreinemachers 2003).

Exposure, for tracking purposes, is defined as the proximity and/or contact with a source of a disease agent in such a manner that effective transmission of the agent or harmful effects of the agent may occur (CDC 2003a). Pesticide exposure surveillance in the United States is largely limited to particular occupational cohorts—medical monitoring of applicators, for example—and to biomonitoring efforts to characterize exposures among representative samples of regional and national populations. In many states, occupational exposures resulting in depressed cholinesterase levels are reportable conditions and are useful for monitoring regulatory compliance, enforcing work rules, managing disease cases, and identifying risk factors (Calvert et al. 2004). But these data have limited generalizability to larger and more varied populations. The U.S. EPA National Human Exposure Assessment Survey, completed in the 1990s, evaluated pesticide exposures among representative populations in Arizona, the Midwest, and Maryland (Berry et al. 2000). This effort has not been replicated. As part of the Third National Health and Nutrition Examination Survey (NHANES III), CDC carried out biomonitoring for metabolites of several classes of pesticides. These data provide, for the first time, baseline exposure estimates for a representative U.S. population to a variety of pesticides (Barr et al. 2004). NHANES III has produced a wellspring of reports based on these results and illustrates how providing exposure data linked to personal descriptors can fill in critical knowledge gaps.

Health effects, for tracking purposes, are chronic or acute health conditions that affect the well-being of an individual or community and are measured in terms of illness and death (CDC 2003a). Although the health effects from pesticides may include acute and chronic conditions and reproductive effects, surveillance of their health impacts is effectively limited to nearly immediate toxic effects. The Toxic Exposure Surveillance System (TESS) is a national surveillance program that collects poison control data from all state and regional poison control centers. TESS records basic hazard, exposure, and individual information on pesticide-related inquiries, of which there were more than 96,000 in 2002 (Watson et al. 2003). Poison Control Center data are useful for identifying educational and outreach needs, identifying risk factors for poisonings, and investigating and identifying clusters and outbreaks. The Sentinel Event Notification System for Occupational Risk (SENSOR) at the National Institute for Occupational Safety and Health (NIOSH) supports pesticide-related illness and injury surveillance in 12 states and is used to identify outbreaks and emergency pesticide health effects (NIOSH 2004).

More than 40 states collect and report hospital discharge data, and pesticide-related hospitalizations are rare. Although more patients report to emergency departments for pesticide exposures than are admitted to hospitals, few states systematically collect and report these data.

## The Case for Urban Pesticide Tracking

The data described above that are systematically collected about pesticide hazards, exposures, and health effects describe the risks experienced by agricultural communities better than those experienced by other groups. There are many reasons, however, why large cities may be interested in developing pesticide tracking systems. A 1999 analysis of New York’s PUR data found that even though NYC accounts for < 1% of the total land area of the state, > 7% by volume of all pesticides applied in the state, and 13% by weight, were applied in NYC. Also, all five counties of NYC were included in the top 10 counties statewide for use of pesticides (Thier 2000).

Several events have elevated the city’s level of awareness about pesticides. Spraying of adulticides for controlling mosquitoes that carry West Nile virus (CDC 2003c), the rise in asthma hospitalizations in the late 1980s through the mid 1990s, the growing awareness of the links between pest infestations and health symptoms, high profile experiments in least-toxic pest control in low-income housing, and public hearings on methods of controlling rats (Kass and Outwater 2002) have all contributed to public concern regarding pesticide health effects. NYC residents have been the subject of several recent studies that have associated negative reproductive health outcomes among low-income women with residential exposure levels to chlorpyrifos (Berkowitz et al. 2003, 2004; Perera et al. 2003; Whyatt et al. 2004). As a result of these events, pesticides have taken on greater importance for public health and housing agencies.

Populations residing in large urban areas face special health risks from a variety of environmental concerns. In NYC and other older, densely populated, largely immigrant cities, environmental hazards tend to concentrate spatially, ethnically, and socioeconomically. Awareness of these hazards may sometimes be great, prompting important and appropriate advocacy and action by communities to ameliorate conditions that contribute to acute and chronic illness. Other times, communities or governmental officials have so little information that speculation, hyperbole, or inaction may result. Under these circumstances, public health agencies play a largely reactive role to public concerns. Failing to unite disparate information on hazards leaves agencies with an incomplete story, and inappropriate policy decisions may result. By linking data sources on pesticide use, housing quality and finance, demographics and socioeconomic status, exposures, and health, much more can be learned about where and why pesticides are used. This deeper understanding may promote the improved targeting of resources, education, and toxic use reduction efforts, as well as inform scientifically sound policy and legislation.

## Materials and Methods

With feedback from a stakeholder advisory panel created to guide the development of the public health tracking program, DOHMH identified seven principles that would guide decision making on data acquisition, data architecture, analytic priorities, and public engagement: The pesticide tracking system should *a*) build upon existing and ongoing data collection systems; *b*) link hazard, human exposure, and human health effects data in scientifically valid and defensible ways; *c*) automate, to the extent possible, the importing, cleaning, and linking of data sources; *d*) build on, rather than duplicate, data and technical systems already under development by data providers; *e*) enable the development and tracking over time of public health environmental indicators; *f* ) satisfy the needs of a wide community of data users, analysts, advocates, and residents; and *g*) inform the development of public health and environmental interventions whose goals are to reduce health risks and improve environmental quality. In this section, we describe preliminary progress toward the creation of NYC’s pesticide tracking system.

### Data sources.

In 2003 the DOHMH, in cooperation with the NYC Department of Information and Telecommunications Technology, began a comprehensive data and metadata inventory of NYC and New York State environmental data. We reviewed data systems at health, housing, finance, planning, and environmental protection agencies for their applicability and relevance to a pesticide tracking system. A metadata database is being populated that includes descriptive information about the data, process information on its collection, contact information, identifiers, geospatial descriptors, system architecture, distribution methods, and anticipated modifications.

Our initial inventory revealed two significant data gaps in the hazard–exposure–outcome tracking triad. First, there is no existing source of data to describe, on a population basis, the exposures of NYC residents to pesticides. Fortuitously, DOHMH’s Division of Epidemiology was already 6 months into its planning for a NYC Health and Nutrition Examination Survey (HANES) when it became clear to the staff of Environmental Connections that by adding pesticide biomonitoring, similar to that carried out in NHANES III, one part of the gap could be closed. In collaboration with CDC’s National Center for Environmental Health Pesticide Laboratory, we plan to collect and analyze urine for organophosphate and pyrethroid metabolites as part of the 2004 NYC HANES. The second gap is a temporary one. Data on emergency department use will first become available in New York in 2005. Until then, we are collaborating with the DOHMH Bureau of Injury Surveillance to abstract charts in 23 emergency departments 1 week each quarter to determine the frequency, scope, and risk factors associated with pesticide poisonings, again opportunistically expanding an existing program for environmental tracking.

Table 1 summarizes results from the data inventory process and identifies the utility of each data source for a pesticide tracking system. In addition to data sources already described, the system will include data from NYC’s annual Community Health Survey, an annual telephone survey of 10,000 city residents, based on CDC’s Behavioral Risk Factor Surveillance System (Karpati et al. 2003). Questions on personal and commercial pesticide applications and cockroach infestations were included in the 2003 questionnaire.

Additional public and commercially available data sets will be linked, including pesticide registration and toxicity data (for grouping and lookup purposes) and Dunn and Bradstreet Business Locator (Providence, RI) (for identifying information on state-registered commercial pest control companies). Table 1 reveals several obstacles in building the tracking system. Hazard, health outcome, and related housing and population data are being acquired from three municipal and three state agencies and from surveys conducted by the U.S. Census Bureau. There is a steep learning curve for researchers to become familiar with the strengths and weaknesses of most large data sets; only some data sets have substantial documentation and data that have been used in published studies. For example, indices of housing disrepair exist and have been validated with housing and vacancy survey data.

For data originally gathered for purposes other than those contemplated here, the task is more difficult. For example, poison control data may have multiple reports of a single incident, redundancies not easily remedied. Building finance data, another example, is a historical data set that maintains all transactions related to parcels. Determining property value from the system’s tax and mortgage records requires algorithms that manage different assessment periods, overlapping loans, and asset transfers into account.

Some data sets may describe different stages of the same incident, such as poison control center, emergency department and hospitalization discharge data. The frequency of update differs among the data sets, posing logistical and methodologic challenges for creating analyzable data sets. Finally, negotiating multiple data use agreements, human subjects assurances, and stakeholder boards is time-consuming and imposes difficult-to-reconcile security requirements on data reporting and public availability.

### Data links.

**Figure 1:**
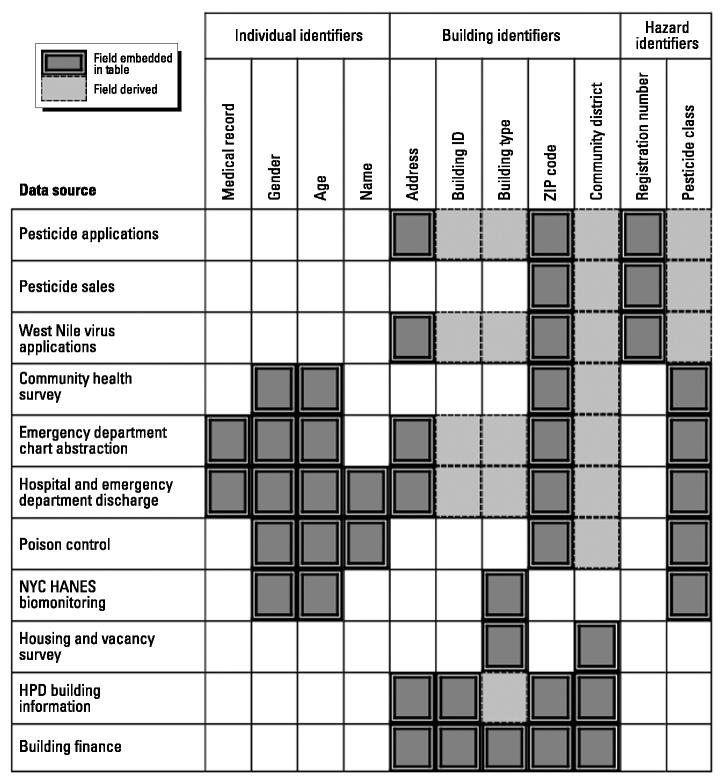
Linkable identifiers among key data sources. HPD, NYC Department of Housing Preservation and Development.

Although each source of data provides useful information for the development of environmental public health indicators, it is the ability to link them that differentiates this effort from simple reporting. Figure 1 describes the individual, building, and hazard identifiers shared among the key data sources for this system. Two types of links are highlighted, embedded, and derived. An embedded link occurs when data fields are shared by two data sets. For example, address data are contained within the PUR applications database and can be directly associated with housing complaint and inspections data in the NYC Department of Housing Preservation and Development data set. A derived link is one made possible through the use of geosupport tools, by the hierarchical nature of the data structure, or via probabilistic matching. For example, once an address is known, a building identification number can be imported into the record using a geosupport system created for NYC. Once a compound’s registration number is known, its pesticide class (e.g., organophosphate) can be determined. If an address is missing from poison control data, then time, age, gender, ZIP code, and other variables can be used to create probabilistic matches to emergency department or hospitalization records.

Figure 1 displays myriad connections among the data sources and can be thought of as a cognitive map of relationships from which hypotheses can be formulated and analyses carried out. The following are some of the questions that can be explored by using these data links:

- Which building-related conditions are associated with the application of pesticides?
- Do hospitalizations reflect the “tip of the iceberg” of health outcomes?
- Is there an association between commercial pesticide applications and biomonitored exposure and type of residential building?
- What is the correlation between reported use of pest-control services in the community health survey and pest control operator-reported applications?
- What are the predictors of the personal use of hazardous pesticides?
- Over time, is the use of pesticide associated with reductions in infestations?

Many methodologic issues confront this analysis. A system with so many sources of data and so many links may yield, by virtue of multiple comparisons, random associations. There are many unresolved issues involved in carrying out geospatial analysis, including the selection of geographic units of analysis, exposure modeling, and determining the potential for exposure, that may dramatically affect findings (Maantay 2002). The quality of some data to be assembled in this tracking system remains largely unknown until additional data sources are gathered and analyzed. Linking data originally gathered for fiscal or regulatory purposes to describe environmental hazards, exposures, and health outcomes raises concerns about the validity of variables, indicators, and indices derived from them.

The system described will also have limited ability to observe associations among hazards and exposures on the one hand, and chronic health outcomes on the other. Neither the hazard nor exposure data necessarily reflect long-term chronic exposures and risks. Poisonings, emergency department visits, and hospitalizations from pesticide-related problems reflect acute conditions resulting from acute exposures. Chronic conditions such as asthma, neurologic disorders, and many cancers may be observed in hospitalization and registry data but cannot be assumed to be related to short-term exposures reflected in the hazard data.

## Discussion

We have completed the initial steps in the identification, acquisition, and assessment of data that can be used to characterize pesticide use, exposure, and health problems in NYC. The system we describe will be built largely on data sources that are pesticide related. Stakeholders are interested not only in the characterization of pesticide hazards, exposures, and poisonings but also in learning more about whether pesticide exposures are associated with Parkinson disease, neurologic disorders, development disabilities, and respiratory health. The potential of pesticide tracking to explore these concerns begins with building a base hazard and exposure system.

The final form, breadth, and analytic strength of this system will depend on many factors—data quality and completeness, the degree of sustained institutional and public support, sufficient funding, and staff resources among them. Despite logistical, resource, and methodologic limitations associated with the development of an urban pesticide tracking system, this system offers the potential for significant benefits for researchers, policy makers, residents, industry, and advocates. A hazard, exposure, and health outcome system has the potential to reveal relationships impossible to assess without linking data sets and to close significant gaps in our knowledge about how, where, when, why, and with what consequences pesticides are used in an urban environment.

## Figures and Tables

**Table 1:** Key data sources for pesticide tracking system.

Data set*^a^*	Managed by	Type of tracking data	Applicability	Update frequency	Required for acquisition
West Nile virus pesticide applications	NYC DOHMH	Hazard	Applications data	Continuous	None
Food pesticide residue	NYS Dept. of of Agriculture	Hazard	Indicators to be tracked	Annual	None
Pest control firm survey	NYC DOHMH	Hazard	Indicators to be tracked	Every 2 years	IRB
Pesticide applications	NYS DEC	Hazard	County/ZIP code applications	Annual	None
			Address-level applications	Annual	Research application, IRB
Pesticide sales	NYS DEC	Hazard	ZIP code–level sales	Annual	None
HPD building information	NYC HPD	Hazard, risk factors	Address-level complaints and violation data	Continuous	Use agreement
Housing and vacancy survey	U.S. Census	Hazard, risk factors	Neighborhood-level housing quality, occupancy	Every 3 years	None
NYC HANES	NYC DOHMH	Exposure, risk factors	Exposure to organophosphates and pyrethroids	Every 3 years	IRB
Community health survey	NYC DOHMH	Hazard, outcome	Neighborhood-level health and pesticide use data	Annual	None
Poison control data	NYC DOHMH	Hazard, outcome	Suspected poisonings	Continuous	Use agreement, IRB
Emergency department chart abstraction	NYC DOHMH	Outcome	Poisoning incidence	Quarterly	None
Hospital and emergency department discharge data	NYS DOH	Outcome	Address-identified outcomes	Annual	IRB
Vital statistics birth records	NYC DOHMH	Population	Intercensus populations	Continuous	Data use agreement
U.S. Census	U.S. Census	Population, risk factors	Fine geography–level demographic, socioeconomic data	Every 10 years	None
Automated city register	NYC Finance	Risk factors	Address-level financial data	Continuous	Data use agreement

Abbreviations: DEC, Department of Environmental Conservation; DOH, Department of Health; HPD, Department of Housing Preservation and Development; IRB, institutional review board; NYS, New York State.

a A list of web site addresses that describe or make available some of these data sets may be obtained by contacting the corresponding author of this article.
